# Supplementary material for: MULTIMODAL NEURAL CORRELATES OF CHILDHOOD PSYCHOPATHOLOGY
Source: bioRxiv. 2024 Aug 17:2023.03.02.530821. Preprint. [Version 2] doi: 10.1101/2023.03.02.530821 (PMC11343159; doi:10.1101/2023.03.02.530821)
Supplement: Supplement 2 [file NIHPP2023.03.02.530821v2-supplement-2.pdf]

bioRxiv preprint doi: https://doi.org/10.1101/2023.03.02.530821; this version posted August 17, 2024. The copyright holder for this preprint (which was not certified by peer review) is the author/funder, who has granted bioRxiv a license to display the preprint in perpetuity. It is made available under aCC-BY 4.0 International license.

## SUPPLEMENTARY FILE CAPTIONS

**Supplementary file 1**. See supplementary files 1a to 1h for supplementary tables and information cited in this study.

## SUPPLEMENTARY FIGURE CAPTIONS

**Figure 1 – figure supplement 1**. Explained covariance by LCs 1-5. They explained 21%, 4%, 3%, 3%, 2% of the covariance between the multimodal imaging data and behavioral data, respectively.

**Figure 1 – figure supplement 2**. Posthoc analyses testing for sex differences in the composite scores between male and female participants. Male participants had higher imaging and behavior composite scores in LC1 (p-factor) and LC3 (neurodevelopmental symptoms), while female participants had higher imaging and behavior composite scores in LC2 (internalizing symptoms).

**Figure 1 – figure supplement 3**. Relative importance of imaging modalities in LCs 1-3. The confidence interval shows the standard deviation across bootstrap samples. RSFC yields higher importance in LC1 compared to structural loadings, while thickness yields lower importance compared to all other modalities in LCs 2-3.

**Figure 2 – figure supplement 1**. Structural loadings associated with LCs 1-3, before FDR correction. FDR-corrected loading maps can be found in Figures 2,3,4.

**Figure 2 – figure supplement 2**. Subcortical volume loadings, and subcortical-cortical FC loadings during resting-state and the three fMRI tasks (Monetary incentive delay, Emotional

bioRxiv preprint doi: https://doi.org/10.1101/2023.03.02.530821; this version posted August 17, 2024. The copyright holder for this preprint (which was not certified by peer review) is the author/funder, who has granted bioRxiv a license to display the preprint in perpetuity. It is made available under aCC-BY 4.0 International license.

n-back, and Stop signal task). Loadings are shown uncorrected (before FDR correction). FDR-corrected loadings can be seen on Figures 2-5.

**Figure 2 – figure supplement 3**. FC patterns during rest and 3 fMRI tasks associated with LCs 1-3, before FDR correction.

**Figure 5 – figure supplement 1**. Principal gradient computed without alignment to the gradients derived from the HCP dataset.

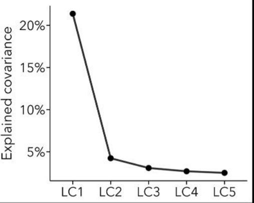

Explained covariance
20%
15%
10%
5%
LC1
LC2
LC3
LC4
LC5

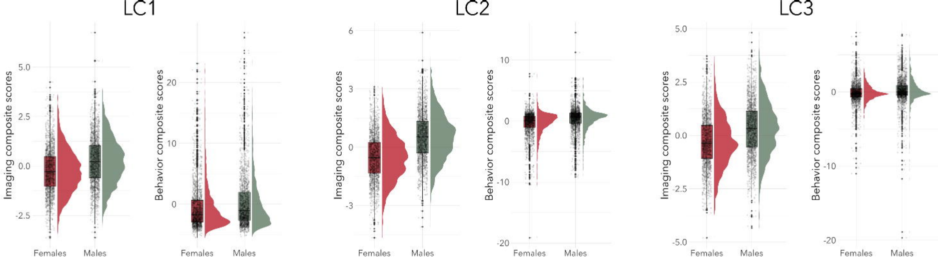

LC1
LC2
LC3
Imaging composite scores
Behavior composite scores
Females
Males
5.0
2.5
0.0
-2.5
20
10
0
6
3
0
-3
10
0
-10
-20
5.0
2.5
0.0
-2.5
-5.0
0
-10
-20

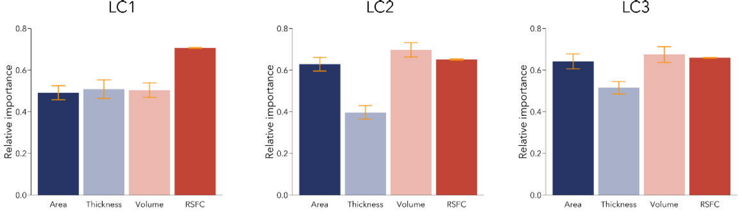

LC1
LC2
LC3
Relative importance
0.0
0.2
0.4
0.6
0.8
Area
Thickness
Volume
RSFC

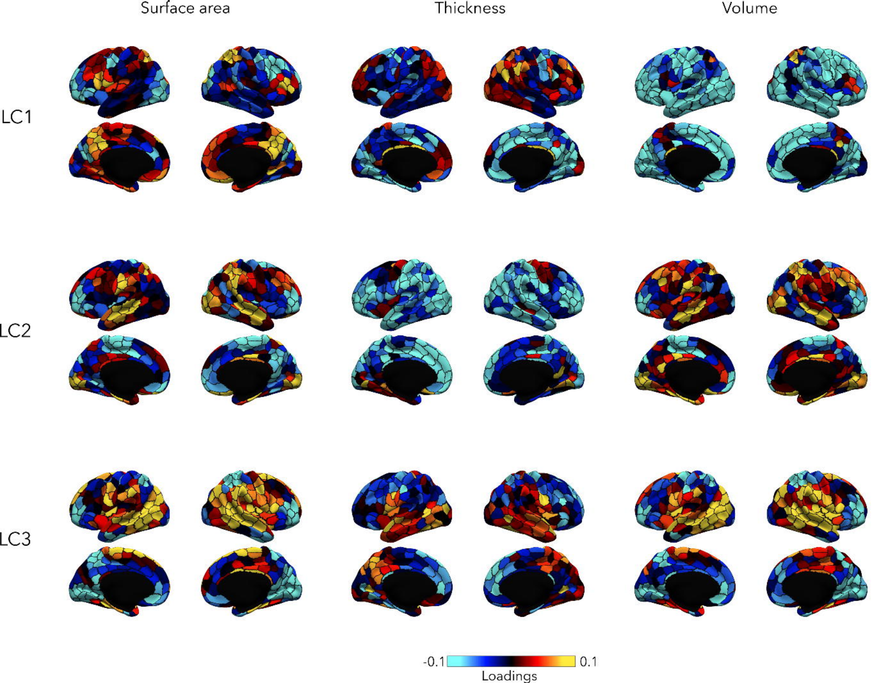

Surface area
Thickness
Volume
LC1
LC2
LC3
-0.1
0.1
Loadings

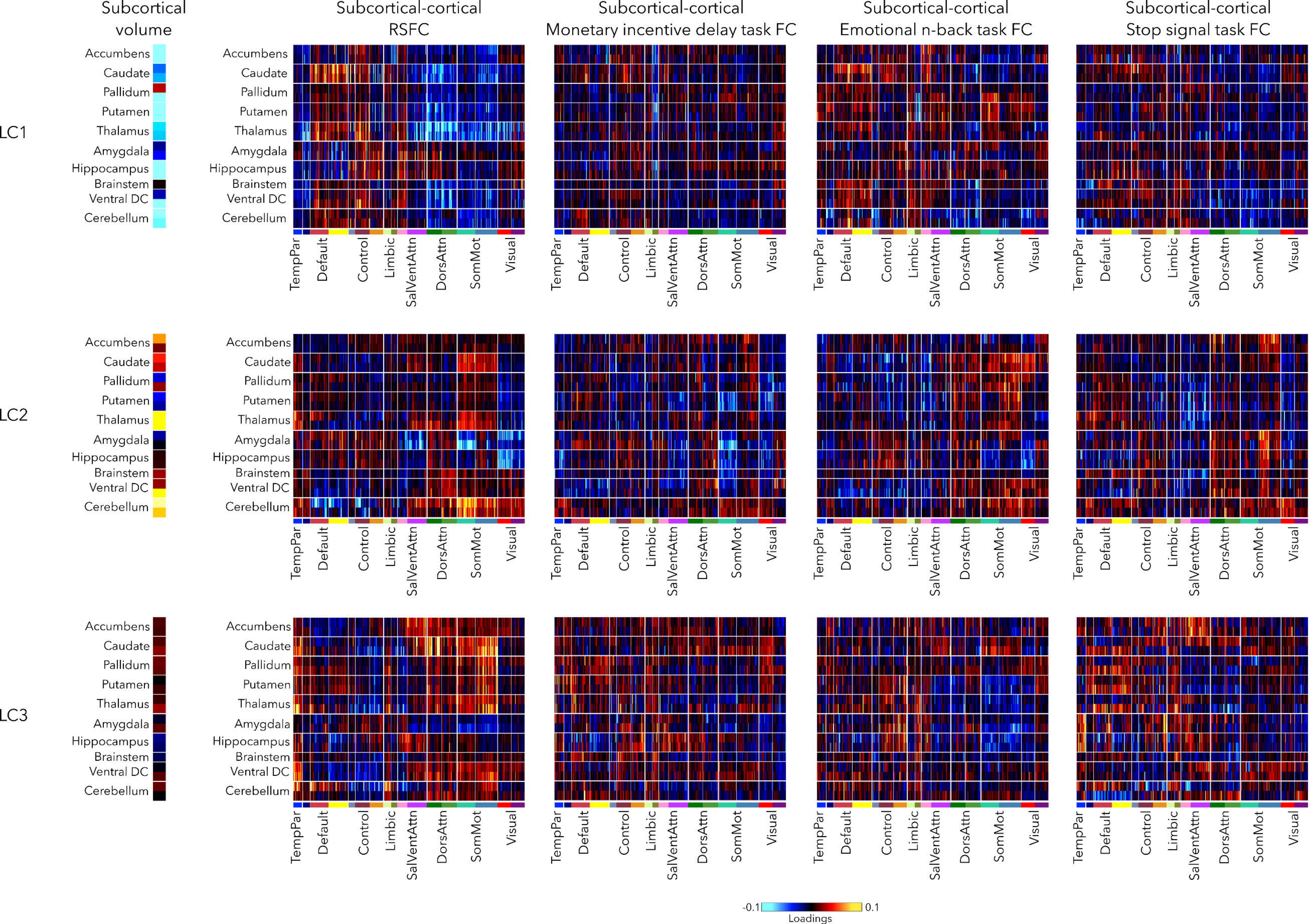

Subcortical volume
Subcortical-cortical RSFC
Subcortical-cortical Monetary incentive delay task FC
Subcortical-cortical Emotional n-back task FC
Subcortical-cortical Stop signal task FC
LC1
LC2
LC3
Accumbens
Caudate
Pallidum
Putamen
Thalamus
Amygdala
Hippocampus
Brainstem
Ventral DC
Cerebellum
TempPar
Default
Control
Limbic
SalVentAttn
DorsAttn
SomMot
Visual
-0.1
0.1
Loadings

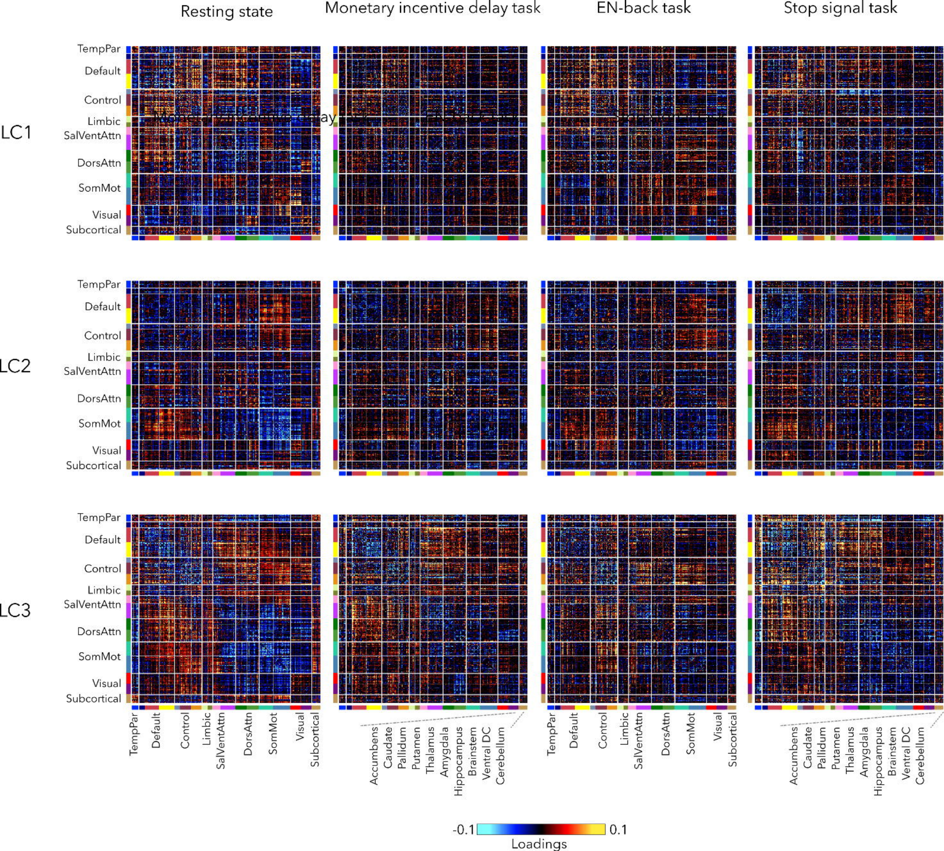

Resting state
Monetary incentive delay task
EN-back task
Stop signal task
LC1
LC2
LC3
TempPar
Default
Control
Limbic
SalVentAttn
DorsAttn
SomMot
Visual
Subcortical
Accumbens
Caudate
Pallidum
Putamen
Thalamus
Amygdala
Hippocampus
Brainstem
Ventral DC
Cerebellum
-0.1
0.1
Loadings

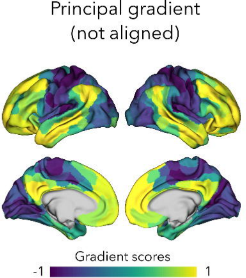
Principal gradient
(not aligned)
Gradient scores
-1
1
